# An alkaline active feruloyl-CoA synthetase from soil metagenome as a potential key enzyme for lignin valorization strategies

**DOI:** 10.1371/journal.pone.0212629

**Published:** 2019-02-25

**Authors:** Victoria Sodré, Juscemácia Nascimento Araujo, Thiago Augusto Gonçalves, Nathália Vilela, Antonio Sergio Kimus Braz, Telma Teixeira Franco, Mário de Oliveira Neto, André Ricardo de Lima Damasio, Wanius Garcia, Fabio Marcio Squina

**Affiliations:** 1 Faculty of Chemical Engineering, University of Campinas (UNICAMP), Campinas, SP, Brazil; 2 Department of Biochemistry and Tissue Biology, Institute of Biology, University of Campinas (UNICAMP), Campinas, SP, Brazil; 3 Centro de Ciências Naturais e Humanas, Universidade Federal do ABC (UFABC), Santo André, SP, Brazil; 4 Programa de Processos Tecnológicos e Ambientais, Universidade de Sorocaba (UNISO), Sorocaba, SP, Brazil; 5 Departamento de Física e Biofísica, Instituto de Biociências, Universidade Estadual Paulista (UNESP), Botucatu, SP, Brazil; Universidade Nova de Lisboa, PORTUGAL

## Abstract

Ferulic acid (FA), a low-molecular weight aromatic compound derived from lignin, represents a high-value molecule, used for applications in the cosmetic and pharmaceutical industries. FA can be further enzymatically converted in other commercially interesting molecules, such as vanillin and bioplastics. In several organisms, these transformations often start with a common step of FA activation via CoA-thioesterification, catalyzed by feruloyl-CoA synthetases (Fcs). In this context, these enzymes are of biotechnological interest for conversion of lignin-derived FA into high value chemicals. In this study, we describe the first structural characterization of a prokaryotic Fcs, named FCS1, isolated from a lignin-degrading microbial consortium. The FCS1 optimum pH and temperature were 9 and 37°C, respectively, with Km of 0.12 mM and Vmax of 36.82 U/mg. The circular dichroism spectra indicated a notable secondary structure stability at alkaline pH values and high temperatures. This secondary structure stability corroborates the activity data, which remains high until pH 9. The Small Angle X-Ray Scattering analyses resulted on the tertiary/quaternary structure and the low-resolution envelope in solution of FCS1, which was modeled as a homodimer using the hyperthermophilic nucleoside diphosphate-forming acetyl-CoA synthetase from *Candidatus Korachaeum cryptofilum*. This study contributes to the field of research by establishing the first biophysical and structural characterization for Fcs, and our data may be used for comparison against novel enzymes of this class that to be studied in the future.

## Introduction

Lignocellulosic biomass harbors a barely explored mine of fermentable sugars and aromatic polymers as structural components. The cellulose and lignin fractions represent the first and second most abundant sources of organic carbon on Earth, respectively, rendering lignocellulose as an attractive possibility for alternative resource of fuels and chemicals [1].

In comparison with first generation biofuels, which utilize sucrose or starch as source of fermentable sugars, the lignocellulosic biomass-reliant second-generation counterparts are considered more cost-effective and less threatening to food security and land usage policies [2]. Nevertheless, the full implementation of second generation biorefineries is hindered by considerable technological and economical hurdles, including feedstock recovery, biomass pretreatment, enzymatic hydrolysis, fermentation optimization and biofuel separation [3,4]. In this context, lignin conversion and valorization has emerged as a possible solution for both cost-effectiveness of second-generation biorefineries and excessive reliance on petroleum-derived chemicals [5–7]. It is predicted that the amount of lignin residue from cellulosic biorefineries solely in the USA will reach as much as 62 million tons per year [8], denoting the great opportunity of valorizing this stream into high-market compounds.

Several research groups are currently focusing in strategies for lignin valorization, mainly exploring chemical catalysis and biological degradation approaches. The latter revolves around “mimicking” nature’s strategies for lignin degradation and consequent carbon cycling, mainly through the identification, characterization and improvement of lignin-degrading organisms and their enzymes. Traditionally, white-rot fungi are the best characterized lignin degraders and there is extensive literature on the enzymatic mechanisms of fungal lignin peroxidases [9,10]. Recently, bacterial degraders have also been the focus of research, not only concerning their capability of lignin depolymerization, but also the catabolism of aromatic molecules derived from its breakdown [11–13]. Therefore, there is great opportunity for genetic and metabolic engineering of microbial pathways, aiming the conversion of lignin in value-added products [14–16]. FA is the precursor of subunit guaiacyl in lignin biosynthesis, is near ubiquitous in plant cells walls of various grasses, where it endows structural rigidity and resistance by cross-linking to pentosan, arabinoxylans and hemicelluloses [17]. As biorefineries adapt to meet the cellulosic ethanol production goals, it is expected that FA, as other cinnamic acid derivatives, will play a major role as resources for bioproducts. Indeed, several studies have demonstrated the successful production of vanillin [18–20] and bioplastics [21] from FA derived from lignin streams and lignocellulosic biomass.

In prokaryotes, FA catabolism occurs via four major pathways: non β-oxidation, β-oxidation, non-oxidative decarboxylation, and side-chain reduction [22]. The first two pathways employ Fcs as initial step, by catalyzing the CoA-thioesterification of FA in feruloyl-CoA, with ATP consumption. The subsequent reactions direct the intermediates towards ring fission by the central metabolism, usually via vanillin and, posteriorly, through the protocatechuate 4,5-cleavage pathway.

Physiologically, this class of enzymes is of particular importance in the sensing of aromatic molecules by microorganisms and activation of respective pathways. The product of the reaction catalyzed by Fcs, feruloyl-CoA, binds to a MarR-family transcriptional repressor and effectively abrogates its ligation to the DNA, thus enabling the expression of genes related to the degradation of FA and other related *p*-hydroxycinnamic acids in several species [23–27]. Recently, the metagenomic profiling of a lignin-degrading consortium, originated from soil of a sugarcane plantation [28], revealed the major metabolic pathways related to degradation of phenolic compounds, including a number of putative sequences correspondent to Fcs, denoting the catabolism of FA and other *p-*hydroxycinnamates.

Currently, the vast majority of scientific articles related to Fcs concentrate on its application for vanillin production, when combined with the action of an enoyl-CoA hydratase/aldolase [29–35]. In addition to that, there is literature focusing on the description of Fcs transcriptional regulatory mechanism and its role in FA degradation [36–39]. Currently, there is no solved structure of prokaryotic Fcs, as well as, a gap of studies analyzing the biophysical properties of this enzyme. In this study, it is presented a comprehensive biophysical characterization and low-resolution envelope, along with the structural stability and biochemical characteristics, of a Fcs, namely FCS1, which was derived from a lignin degrading consortium.

## Materials and methods FCS1

### Sequence and architecture analysis

FCS1 sequence (GenBank accession: MG214406) was retrieved, codon-optimized, synthetized and cloned as previously described [28]. This previous work also showed the enzyme applicability for bioproduction of vanillin, together with an enoyl-CoA hydratase/aldolase. The domain architecture of the enzyme, named FCS1, was further evaluated using the Pfam and SMART databases [40–42]. Calculation of biochemical and biophysical parameters for the putative protein sequence were performed via Protparam [43] online tool. The annotation of orthologous groups was done by comparison against the EggNOG 4.5.1 database [44]. The phylogenetic tree was built with MEGA 7 [45].

### Expression and purification of recombinant FCS1

The cells from a single colony of *E*. *coli* BL21 (DE3) containing the construction pET28a-FCS1 were grown in liquid LB medium supplemented with kanamycin (50 μg/mL) for 16 h at 37°C and 200 rpm. 8 mL of the overnight culture were used to inoculate 800 mL of fresh LB-kanamycin medium, followed by incubation at 37°C and 200 rpm until the optical density (OD) at 600 nm reached a value of 0.6–0.8. To induce recombinant protein expression, isopropyl β-D-1-thiogalactopyranoside (IPTG) was added to the culture to a final concentration of 0.5 mM, followed by incubation at 30°C, 200 rpm for 4 h. After expression, the culture media was centrifuged at 10,000 rpm, 4°C for 15 min and the cell pellet was kept at -20°C.

The cell pellet was resuspended in buffer A (20 mM sodium phosphate buffer pH 7.0, 100 mM NaCl, 5 mM imidazole) containing 0.3 mg/mL of lysozyme, 1 mM of DNase and 5 mM of phenylmethane sulfonyl fluoride (PMSF). Following incubation at room temperature for 1 h, under agitation, the cells were disrupted in ice bath by an ultrasonic processor (10 pulses of 30 s at 30% duty cycle; Ultrasonic Homogenizer 4710 Series, Cole-Palmer Instruments). The soluble protein fractions were obtained by centrifugation for 1 h and 30 min at 8,000 rpm, 4°C, and the resulting supernatant was filtered once in 0.45 μm and twice in 0.22 μm filters. The final solution containing the protein of interest was purified by affinity chromatography in AKTA Start system (GE Healthcare, Waukesha, WI, USA) using a 5 mL HiTrap Chelating HP column (GE Healthcare) charged with Co^2+^ and pre-equilibrated with buffer A. Elution of FCS1 was achieved using a 0–100% linear gradient of buffer B (20 mM sodium phosphate buffer pH 7.0, 100 mM NaCl, 500 mM imidazole). Afterwards, the eluted protein was further purified by size-exclusion chromatography using a Superdex 200 HiLoad 16/600 GL column (GE Healthcare) in buffer C (20 mM sodium phosphate buffer pH 7.4, 100 mM NaCl). Buffer Exchange and protein concentration were performed using Amicon device (Merck, USA). The concentration of purified FCS1 was measured by DeNovix DS-11 spectrophotometer (ε_280nm_ = 43,570 M^-1^cm^-1^) (Uniscience, USA) and by Bradford quantification [46]. Sodium dodecyl sulfate-polyacrilamide gel electrophoresis (SDS-PAGE) was performed using a 12.5% gel in a Mini-PROTEAN Tetra System electrophoresis cell (Bio-Rad, Hercules, CA, USA). The gel was stained with Coomassie brilliant blue R250 for 3 h and distained in distilled water overnight.

### Enzymatic assay

The enzymatic assays were adapted from previously described methods [47,48], the schematic reaction was included in S1 Fig. The reaction mixture (200 μL) contained 2.5 mM of MgCl_2_, 0.5 mM of FA, 2.0 mM of ATP, 0.4 mM of coenzyme A, 40 ng of purified FCS1 and 100 mM of potassium phosphate buffer pH 7.8. The activity assay was initiated by the addition of the enzyme and incubated at 37°C for 10 min. Following incubation, 150 μL of the reaction were used to read the absorbance at 345 nm due to formation of feruloyl-CoA (ε_345nm_ = 1.9 x 10^4^ M^-1^cm^-1^), using an Epoch2 Microplate Reader spectrophotometer (BioTek, Winooski, VT, USA).

For determination of kinetic parameters of FCS1, the above reaction was assayed with substrate concentrations ranging from 0.05 mM to 0.50 mM. Mathematical adjustments were made using the software Graph Pad Prism 5.0 (GraphPad Software) to calculate the parameters. The assays were performed in triplicate and at least three independent experiments were carried out.

### Influence of pH and temperature in enzymatic activity and stability

The temperature range from 15°C to 90°C was chosen for determination of FCS1 optimal temperature. A total volume of 200 μL reactions (as described above) were incubated at the temperature range in a T100 thermocycler (BioRad, Hercules, CA, USA) for 5 min, using the assay do conditions described above.

The enzymatic activity in a range of pHs was assayed in either 100 mM potassium phosphate buffer (pHs 5.8–8.0) or 20 mM (acetate-borate-phosphate) buffer (pHs 7.4–9.4). After stopping FCS1 reaction by heat inactivation (70°C for 15 min), the was pH neutralized to 7.8 by adding a highly ionic strength buffer (phosphate buffer at 2 M). Then, the volume of 150 μL of each reaction were collected and absorbance at 345 nm was read immediately. As controls, reactions without added enzyme were used for each temperature and pH value evaluated. All reactions and controls were made in triplicate and the average values reported. All initial assays were conducted with an additional control containing FA and heat-inactivated enzyme.

For thermostability determination, purified FCS1 in 100 mM potassium phosphate buffer pH 7.0 was incubated at room temperature, 25, 37, 45, 70 and 85°C for 15, 30 and 60 min. For evaluation of enzyme stability in different pHs, 400 ng of purified FCS1 was incubated for 1, 5 and 24 h in 20 mM ABF buffer, pH 7.0–10.0, at 4 ºC. Residual activity was assayed as described in the enzymatic assay topic (incubation at 37°C for 10 min) and immediately read at 345 nm.

### Molecular modeling and conservation analysis

The servers for remote protein homology detection HHPRED [49] and PHYRE2 [50] were used to search for homologs for FCS1 in the Protein Data Bank (PDB) [51]. Molecular model for FCS1 was built using homology-modeling methods and the MODELLER software [52]. The alignment of the amino acids sequence of FCS1 against the amino acids sequence of NDP-forming acetyl-CoA synthetase from the hyperthermophilic archaeon *Candidatus Korachaeum cryptofilum* (*ckc*ACD) [53] was used as input to the MODELLER software, together with the atomic coordinates of the latter (PDB 4XYL, dimeric structure) used as template. The homology model was modeled as a dimer. The template (PDB 4XYL) has 4 chains (A, B, C and D). The chains A and B served as templates for the chain A of our model (monomer), while the chains C and D served as templates for the chain B of our model. Sequences were aligned with Protein BLAST [54]. Conservation analysis was conducted with T-Coffee [55] software using 1.002 sequences with E-value < 1e-100.

### Dynamic Light Scattering (DLS)

The hydrodynamic radius (R_H_) of the FCS1 was determined by dynamic light scattering (Zetasizer, Malvern Instruments Ltd, Malvern, UK). This system employs a fixed scattering angle of 173^o^. DLS measurements for FCS1 were performed at 1 mg/mL in 20 mM Tris-HCl buffer adjusted at pH 7. The hydrodynamic radius was converted to molecular mass (kDa) considering a spherical molecule and using the Zetasizer software.

### Small-angle X-ray scattering (SAXS) data collection and analysis

The SAXS measurements were performed at the SAXS beamline of National Synchrotron Light Laboratory (LNLS, Campinas, Brazil). SAXS measurements for FCS1 were performed at 1 and 5 mg/mL in 20 mM Tris-HCl buffer adjusted at pH 7. The samples were centrifuged at 16,000xg for 10 min (at 4 ^o^C) and then loaded into a 1 mm path length cell made of two thin parallel mica windows and maintained at 20 ^o^C during the measurements. The wavelength of the X-ray beam was 1.48 Å and the sample-to-detector distance (1 m) was adjusted to record the scattering intensity for q values ranging from 0.013 to 0.340 Å^-1^. A total of five successive frames of 60 s were recorded for each sample and buffer scattering was measured before each sample scattering. The X-ray patterns were recorded employing a two-dimensional CCD detector (MarResearch, USA) and the integration were performed by the FIT2D software [56]. The scattering of water measured on the same sample cell was used to normalize the data to absolute scale. The distance distribution function was evaluated using the GNOM software [57]. At least eight dummy atom models (DAMs) were calculated using the DAMMIN software [58] without imposing any symmetry restrictions, and the resulting models were compared with each other using the DAMAVER software [59]. The CRYSOL software [60] was used to generate theoretical scattering curves from the three-dimensional structures. The atomic coordinates of the low-resolution model of FCS1 are available from the corresponding author upon request.

### Circular dichroism (CD) spectroscopy

The CD spectra were measured using a Jasco J-815 spectropolarimeter. FCS1 concentration was 10 μM (0.75 mg/mL) in 20 mM acetate–borate–phosphate buffer adjusted at different pH values (pH 3, 4, 6, 7, 8, 9 and 10). FCS1 was incubated overnight in the corresponding buffer prior to measurements. All spectra were measured using a 1 mm quartz cuvette at 20 ^o^C over the wavelength range from 190 to 270 nm. For each pH value, eight scans (obtained on degree scale, mdeg) were averaged to form the final CD spectrum. Furthermore, CD measurements were collected at pH 7 and different temperature values (20, 60, 70 and 90 ^o^C). The buffer contribution was subtracted in each of the experiments.

### Fluorescence spectroscopy

The Fluorescence emission measurements were performed on a steady-state spectrofluorometer model Cary Eclypse Varian. FCS1 concentration was 5 μM (0.38 mg/mL) in 20 mM acetate–borate–phosphate buffer adjusted at different pH values (pH 3, 4, 6, 7, 8, 9 and 10). FCS1 was incubated overnight in the corresponding buffer prior to measurements. The three tryptophan residues of FCS1 were excited at 295 nm (20 ^o^C) and the fluorescence emission was measured from 300 to 450 nm. The buffer contribution was subtracted in each of the experiments. The measurements were made in triplicate and the average values reported.

### Electrophoretic light scattering (ELS)

The ELS measurements were performed on a Zetasizer Nano ZS (Malvern) [61,62]. The measurements were performed using a fixed FCS1 concentration of 13.4 μM (1 mg/mL) in 20 mM acetate–borate–phosphate buffer adjusted at different pH values. FCS1 was incubated overnight in the corresponding buffer prior to ELS measurements.

## Results and discussion

### FCS1 sequence has three conserved domains

**Fig 1:**
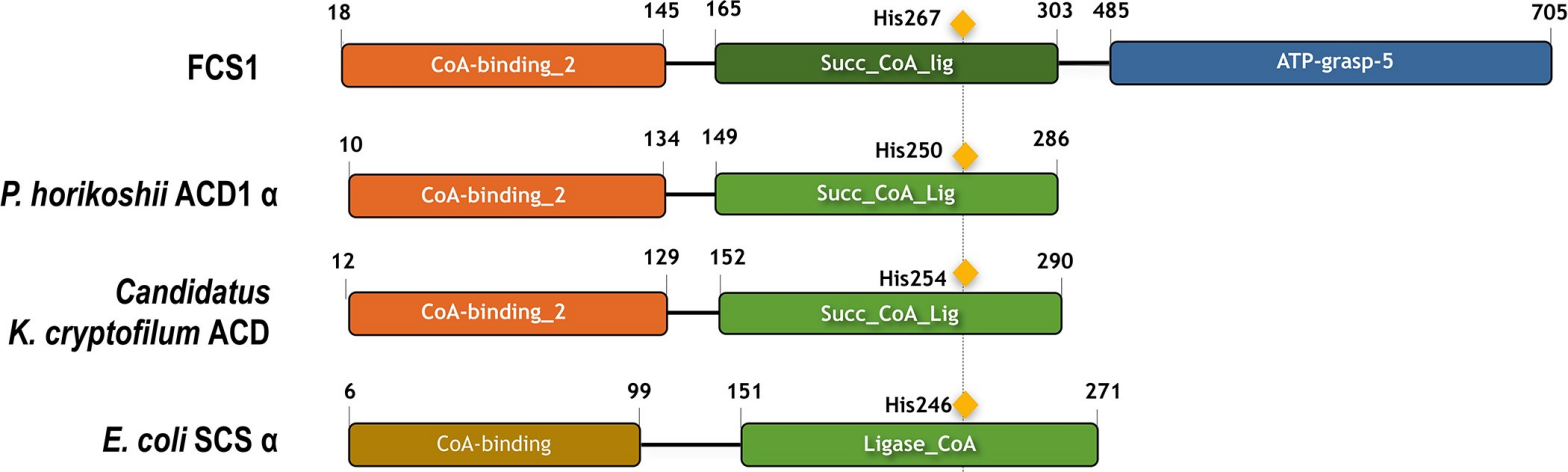
FCS1 domain architecture. FCS1 domain architecture compared against two closest homologues with defined structure from *Pyrococcus horikoshii* (2CSU); *Candidatus Koracheaum cryptofilum* (4XYL); and *E*. *coli* (1SCU_A), which is related protein succinyl-CoA synthetase, alpha chain. The conserved catalytic histidine residue is highlighted in the domain architecture. PFAM domains: CoA binding-2, PF13380 (E-value = 8e-23); succynil-CoA like ligase flavodoxin, PF13607 (E-value = 1e-40); ATP grasp_5, PF13549 (E-value = 9e-58).

The domain architecture of FCS1 is characteristic of the superfamily of nucleoside diphosphate-forming (NDP-forming) acyl-CoA synthetases, which includes other enzymes that catalyze the CoA-activation of acids in corresponding thioesters, such as ATP citrate lyases, pimeloyl-CoA synthetases and maloyl-CoA synthetases (Fig 1). Notably, this superfamily also includes members that catalyze the opposite reaction, through conversion of CoA-thioesters in acids, coupled with substrate-level phosphorylation (e.g. succinyl-CoA synthetases and NDP-forming acetyl-CoA synthetases) [63].

The CoA-binding proteins catalyze a myriad of reactions, reflecting the wide array of CoA binding modes observed in nature [64]. In the case of FCS1, the CoA-binding domain presents a Rossmann fold, which has been shown to bind the 3’-phosphate moiety of the CoA molecule in *E*. *coli* succinyl-CoA synthetase alpha chain (SCSα, PDB: 1SCU_A) [65]. The same conformation was observed in *ckcACD*, an NDP-forming acetyl-CoA synthetase with known crystallographic structure, from the hyperthermophilic archaeon *ckc*ACD (PDB: 4XYL_A). Both examples (*ec*SCSα and *ckc*ACD) display the thiol group of the CoA molecule pointed towards a conserved histidine residue, located in the CoA-ligase domain. This residue is also conserved in FCS1 (His267) and has been shown, in *ec*SCSα, to be transiently phosphorylated during the course of the reaction, therefore acting as an intermediate step between ADP + P_i_ and ATP. The conserved phospho-histidine residue is also present in FCS1 closest orthologues and homologues, as shown in Fig 1.

The comparison of FCS1 amino acids sequence against the NCBI-nr database, using the BLASTp Suite alignment tool, showed high sequential identity values with a CoA-binding protein from *Altererythrobacter sp*. Root672 (89% identity, accession number WP_055917331.1), *Altererythrobacter atlanticus* (88% identity, accession number WP_046904613.1) and Sphingomonadales bacterium 12-68-11 (88% identity, accession number OYW44890.1). The aromatic-degrading potential of members from the Sphingomonadales family has been previously described in the literature, notably *Sphingobium sp*. SYK-6 [24,38] which has a relatively well-characterized FA metabolism. In addition, the *Altererythrobacter* genus has been shown to populate halotolerant lignocellulose degrading microbial consortia [66] and also to be able to degrade petroleum-derived aromatics [67,68].

### FCS1 is a mesophilic alkaline enzyme

**Fig 2:**
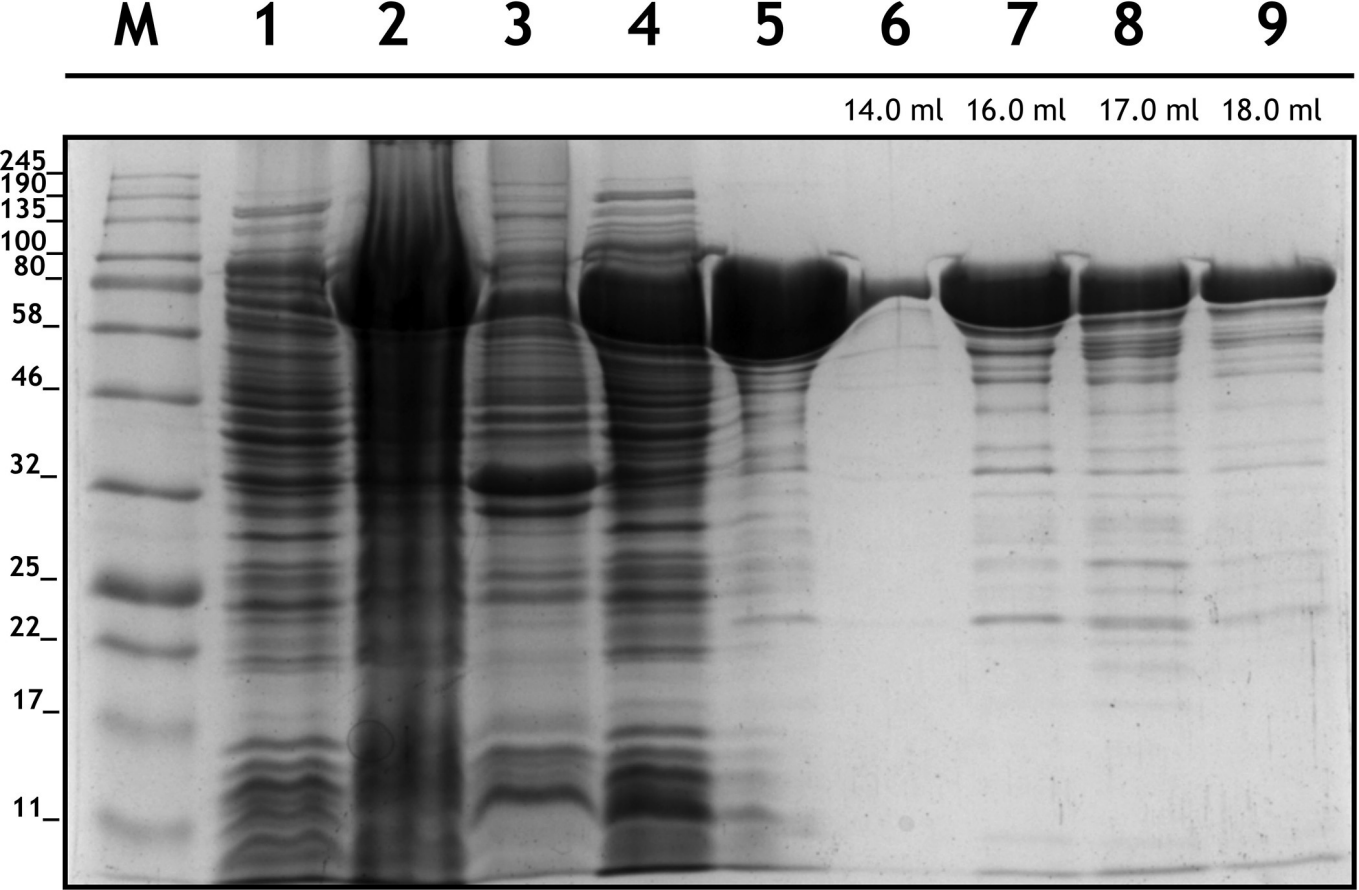
SDS-PAGE gel depicting FCS1 expression and purification steps. Lane M, Molecular Marker (Color Prestained Protein Standard, Broad Range, NEB); lane 1, culture supernatant fraction immediately after IPTG addition; lane 2, cell pellet after 4 hours of IPTG-induced expression (before lysis for purification); lane 3, leftover cell pellet after lysis; lane 4, crude extract (35 μg); lane 5, affinity chromatography fraction (10 μg); lanes 6 (2 μg), 7 (12 μg), 8 (5 μg) and 9 (3 μg) size-exclusion chromatography fractions (elution volumes are shown). See in the supplementary material the metal affinity and size-exclusion column chromatograms (S2 Fig).

The coding sequence of FCS1 was cloned into pET-28a(+), in fusion with a His_6_-tag sequence, for heterologous expression in *E*. *coli* BL21(DE3). The presence of the N-terminal His_6_-tag allowed purification by affinity chromatography, which was followed by an extra purification step by size exclusion chromatography (SEC). In Fig 2, an intense band corresponding to the expressed FCS1 can be observed at approximately 80 kDa, which agrees with the predicted molecular mass of 74.9 kDa.

**Fig 3:**
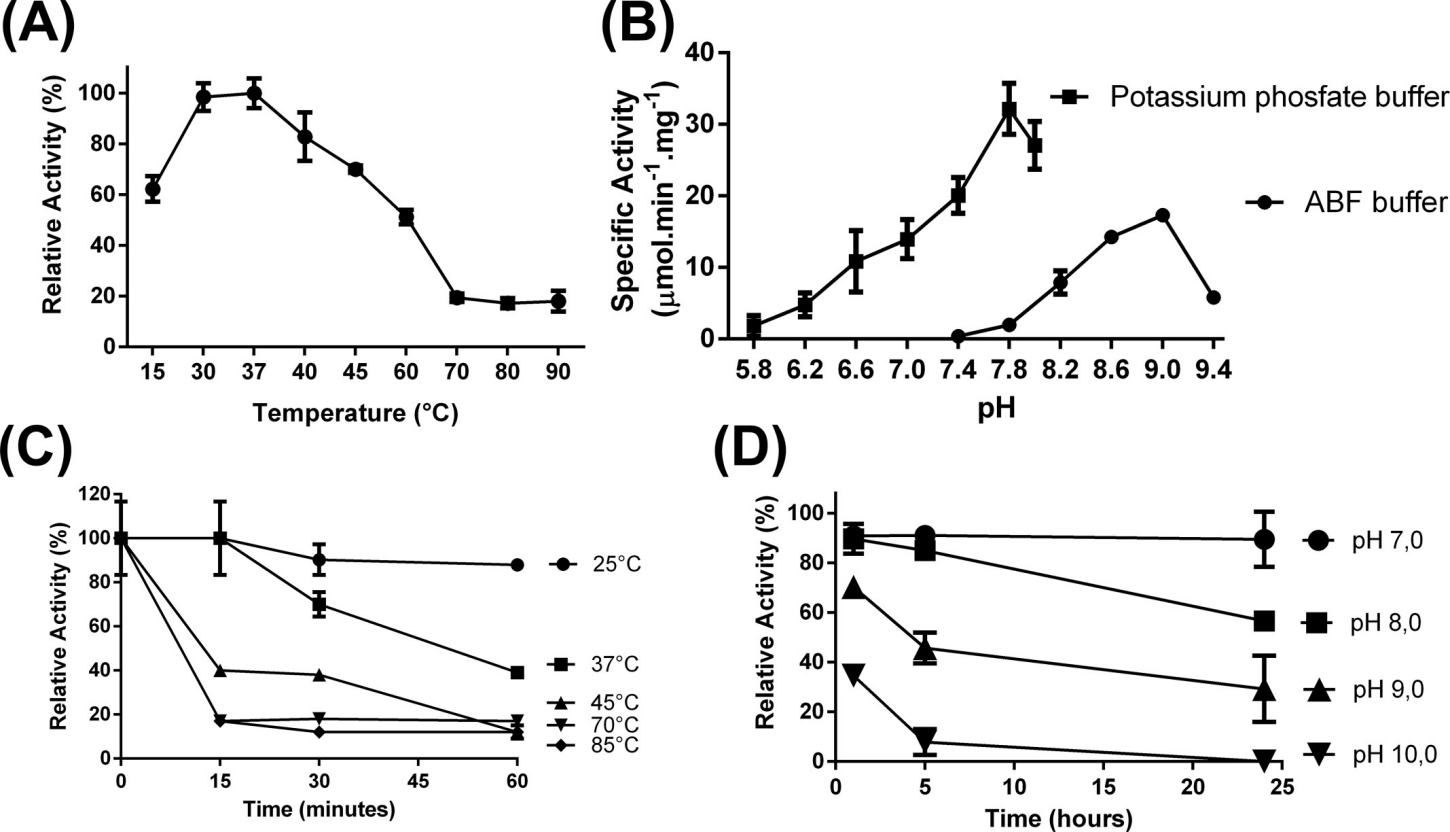
**Optimum temperature (A), pH (B), thermostability (C) and pH-stability for FCS1 (D)**.

The Fig 3B represents the optimum pH for FCS1 enzymatic activity. In 100 mM potassium phosphate buffer, the FCS1 showed poor activity in acidic conditions, which increased rapidly from pH 7.0 onwards, reaching its maximum at pH 7.8. Using the ABF buffer, the FCS maintained accentuated enzymatic activity at alkaline conditions until pH 9. The high enzymatic activity at alkaline pH was further confirmed by capillary zone electrophoresis, depicting the appearance of feruloyl-CoA as product of reaction (S3 Fig). The stability of enzyme activity under alkaline conditions was also monitored after incubation for 1, 5 and 24h in ABF buffer at pH 7.0, 8.0, 9.0 and 10.0 (Fig 3D). Remarkably, the relative activity remained as high as 60% and 40% after prolonged incubation (24 h) in pH 8.0 and 9.0, respectively. This feature could be useful for conversion of lignin streams derived from biorefineries; for instance, Brenelli et al described a fractionation process based on acidification steps, starting from lignin derived alkaline treatment of steam-exploded sugarcane bagasse, that resulted on soluble fractions of pH 8.0 and 9.0 containing FA [69]. According the study, the pH of soluble fractions containing FA show not only pH 8.0 (9.0) but also pH 4.0, 6.0, and 10.0 [69].

It is important to mention that is advisable caution when spectroscopically assaying Fcs at alkaline pH values. The UV-spectroscopy characteristics of FA and related compound are influenced by the nature and pH of the solvent system [70]. Indeed, as can be appreciated in S4 Fig, there is a bathochromic shift of the FA spectra from pHs 8.6 to 10.0. Friedman and Jürgens [71] have demonstrated that FA remains stable in alkaline pHs and suggested that the pH-induced change in FA λ_max_ could be due to an equilibrium between ionized (pH 9) and non-ionized (pH 8) species. According to our data, the optimum pH determined in ABF buffer was 9, which is close to the pK value of FA determined by Friedman and Jürgens. Similarly, the 4-hydroxycinnamoyl-CoA hydratase/lyase from *Pseudomonas fluorescens* AN103, which uses feruloyl-CoA as substrate, showed an optimum pH value at or close to phenolic ionization values for 4-hydroxycinnamoyl-CoA thioester [72]. The lower detectable FCS1 activity in ABF buffer, compared to phosphate buffer, could be due to buffer influence, rather than pH-related. It has been shown that different buffer systems could preferentially inhibit or boost activity in the same pH. This was first observed in cinnamoyl-CoA synthetases by Gross and Zenk [48], which demonstrated the pH optimum in potassium phosphate buffer pH 6.7, whilst the same pH in Tris-HCl exhibit activity 70% lower. Similar results were observed for the activity of an ω-hydroxypalmitate *O*-feruloyl transferase from potato [73], which catalyzes the transfer of FA from feruloyl-CoA to 16-palmitic acid: in comparison to potassium phosphate buffer pH 7.0, activity at the same pH in Tris-HCl buffer was approximately 40% lower.

The Fig 3A shows the optimum temperature for FCS1 enzymatic activity. The FCS1 optimum temperature was 37°C, although nearly 100% activity can be obtained at values between 30°C and 37°C. This is in agreement to the optimum temperature determined for other two previously characterized feruloyl-CoA synthetases, isolated from *Streptomyces* sp. V-1 [35] and *Pseudomonas putida* [48]. The FCS1 retains approximately 60% activity in 15°C. Relative activity steadily decreases from 37°C upwards, reaching a minimum of 20% at 70°C, which remains stable up to 90°C.

The FCS1 thermostability (Fig 3C) was determined by incubating the enzyme at 25, 37, 45, 70 and 85°C for up to 60 min. FCS1 activity remained high and stable at room temperature (25°C) during all time points (15, 30 and 60 min); at 37°C residual activity remained maximal up to 30 min of incubation and it steadily decreased reaching approximately 60% of residual activity after 60 min of incubation. At 45°C, FCS1 residual activity was reduced to 50% after 15 min of incubation and decreases at a regular rate until reaching a minimum of 20% after 60 min.

The specific activity of FCS1 was spectrophotometrically assayed and calculated based on the molar extinction coefficient for feruloyl-CoA, that was first determined by Gross and Zenk (ε_345nm_ = 1.9 x 10^4^ M^-1^ cm^-1^) [47], rendering a value of 30.8 ± 0.8 U/mg. The FCS1 displayed classical Michaelis-Menten kinetics (S5 Fig) the *Km* and Vmax values were calculated as 0.1 mM and 36.8 U/mg, respectively. The catalytic constant (*kcat*) is 45.9 s^-1^ and the catalytic efficiency (*Kcat*/*Km*) is 371.6 mM^-1^ s^-1^. Table 1 compares the biochemical characteristics of FCS1 and several previously described prokaryotic feruloyl-CoA synthetases.

**Table 1:** Comparison of biochemical parameters from several prokaryotic feruloyl-CoA synthetases.

Organism of origin	Enzyme preparation	Detection of activity	Specific activity (U/mg)	Km (mM)	Vmax (U/mg)	Kcat/Km (mM ^-1^. s^-1^)	Optimum pH	Optimum Temperature (°C)	Reference
Unknown (lignin consortium)	Heterologous expression in *E*. *coli* and purification	Optical assay*/capillary electrophoresis	30.7± 0.8	0.1	36.8	371.6	7.8	37	This work
*Streptomyces* sp. V-1	Heterologous expression in *E*. *coli* and purification	Optical assay**/GC	70.6	0.4	78.2	193.4	7.0	30	[35]
*Rhodococcus jostii* RHA1	Heterologous expression in *E*. *coli* and purification	RP-HPLC/LC-MS	-	0.1±0.0	-	160.0	-	-	[39]
*Pseudomonas putida*	Expression and purification from *P*. *putida*	Optical assay*	6.1	75	0.4	-	6.7	37	[48]
*Amycolatopsis* sp. HR167	Soluble fraction of crude cell extracts	Optical assay**/HPLC	105	-	-	-	-	-	[36]
*Pseudomonas* sp. HR199	Soluble fraction of crude recombinant *E*. *coli* cell extracts	Optical assay**/HPLC	0.2	-	-	-	-	-	[37]
*Sphingomonas paucimobilis* SYK-6	Soluble fraction of crude recombinant *E*. *coli* cell extracts	Optical assay*/GC-MS	0.1± 0.0	-	-	-	-	-	[38]
*Pseudomonas fluorescens* AN103	Soluble fraction of crude cell extracts	Optical assay*	5.8	-	-	-	-	-	[74]

*Optical assay quantification done as described by Zenk and Gross [49], with ε_345nm_ = 1.9 x 10^4^ M^-1^cm^-1^.

**Optical assay quantification using ε_345nm_ = 10 cm^2^ μmol^-1^.

-: not determined/reported.; The error values are shown when available in the cited manuscript.

### FCS1 forms stable homodimers in solution

**Fig 4:**
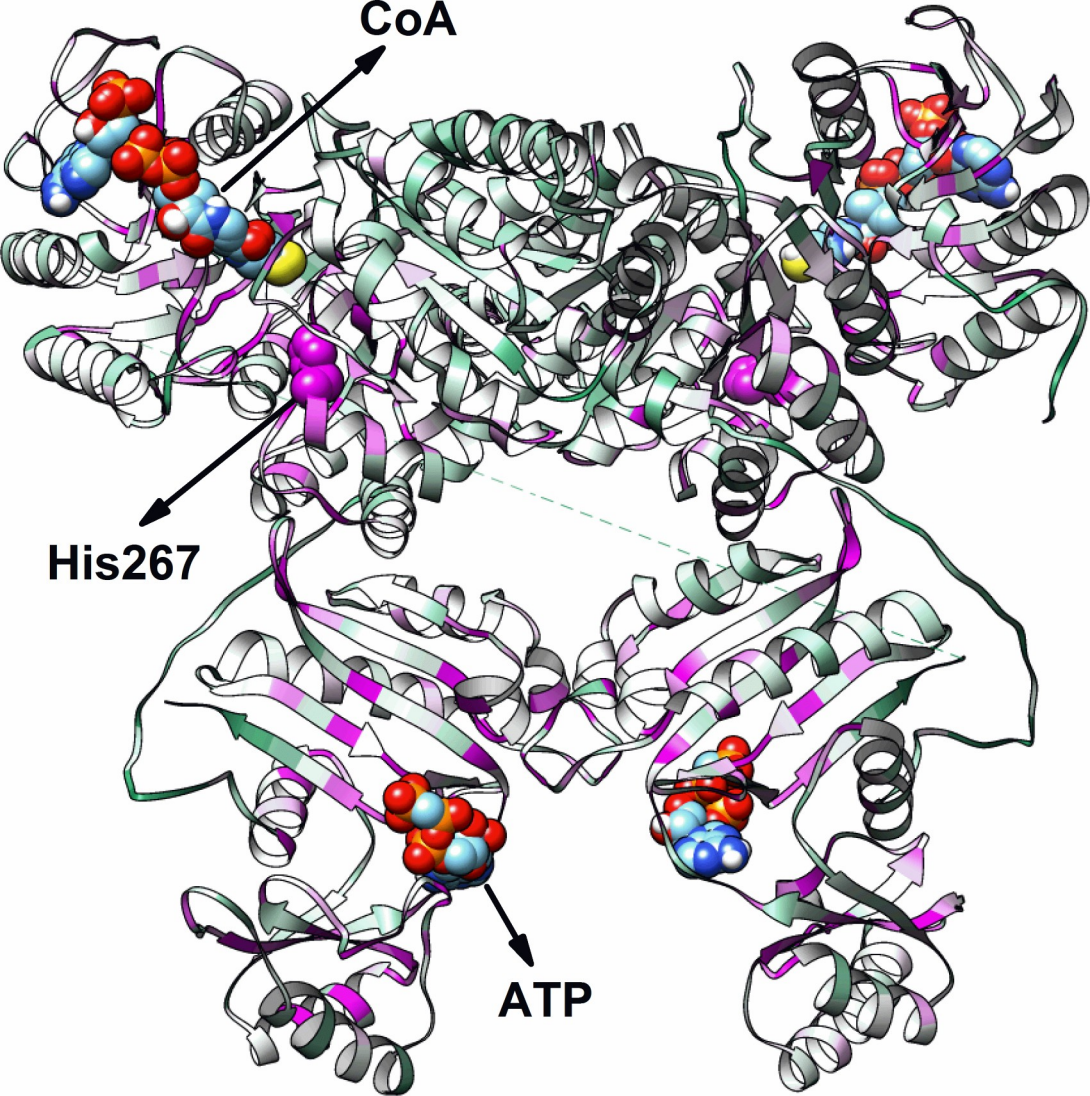
FCS1 homology model. N-terminal is composed by a Rossmann-like fold domain (CoA binding domain) followed by two domains with a flavodoxin-like fold, while the C-terminal corresponding to an ATP-grasp fold where is localized the ATP-binding site. CoA and ATP molecules are indicated in the figure and represented by colored spheres. The conserved residue His267 is represented by pink spheres. Structural distribution of conservation. Conservation degrees were converted into color gradients: pink—highly conserved (> 90%); white—partially conserved; green—little conserved (< 10%). Highly conserved residues (pink) are concentrated around the CoA and ATP molecules as well as around the conserved residue His267.

The three-dimensional structure of the FCS1 was modeled using bioinformatics tools (Fig 4). The threading methods used indicated that FCS1 presents low sequence identity (35%) when compared with NDP-forming acetyl-CoA synthetase from the hyperthermophilic archaeon *ckc*ACD, however it indicated high level of confidence homology (95%) which is a very strong indicator that FCS1 adopts a highly similar fold to that reported for *ckc*ACD.

After nickel-affinity column, the recombinant FCS1 was applied to a size-exclusion column (SEC) (S2 Fig). The FCS1 appeared as a single peak in the elution profile with a molecular mass greater than 105 kDa (the largest molecular mass standard used in the experiment; see S6 Fig), indicating that the protein forms oligomers in solution, as the expected molecular mass for FCS1 is 75 kDa. Furthermore, when FCS1 was analyzed by DLS in solution, the observed profile was characteristic of a monodisperse protein in solution (S6 Fig). The value of hydrodynamic radius determined for FCS1 was 5.2 ± 0.2 nm. This value corresponds to an estimated molecular mass of 160 ± 15 kDa, considering a spherical molecule in solution, consistent with a homodimer for FCS1 in solution.

Therefore, FCS1 was modeled as a homodimer using *ckc*ACD crystallographic structure (PDB 4XYL) as template. The homology model obtained for FCS1 monomer showed two different regions. The N-terminal region is composed of three distinct domains with a mixture of α and β secondary structures: a domain with a Rossmann-like fold [75] (CoA binding domain) followed by two domains with a flavodoxin-like fold [76]. The C-terminal region corresponding to an ATP-grasp fold where is localized the ATP-binding site [77]. The final domain disposition obtained by 3D-modelling is in accordance to the architecture predicted by comparison against the Pfam and SMART databases. In agreement to the homology model, several highly conserved residues (highlighted in pink) are concentrated around the CoA and ATP molecules, as well as, around the conserved residue His267.

**Fig 5:**
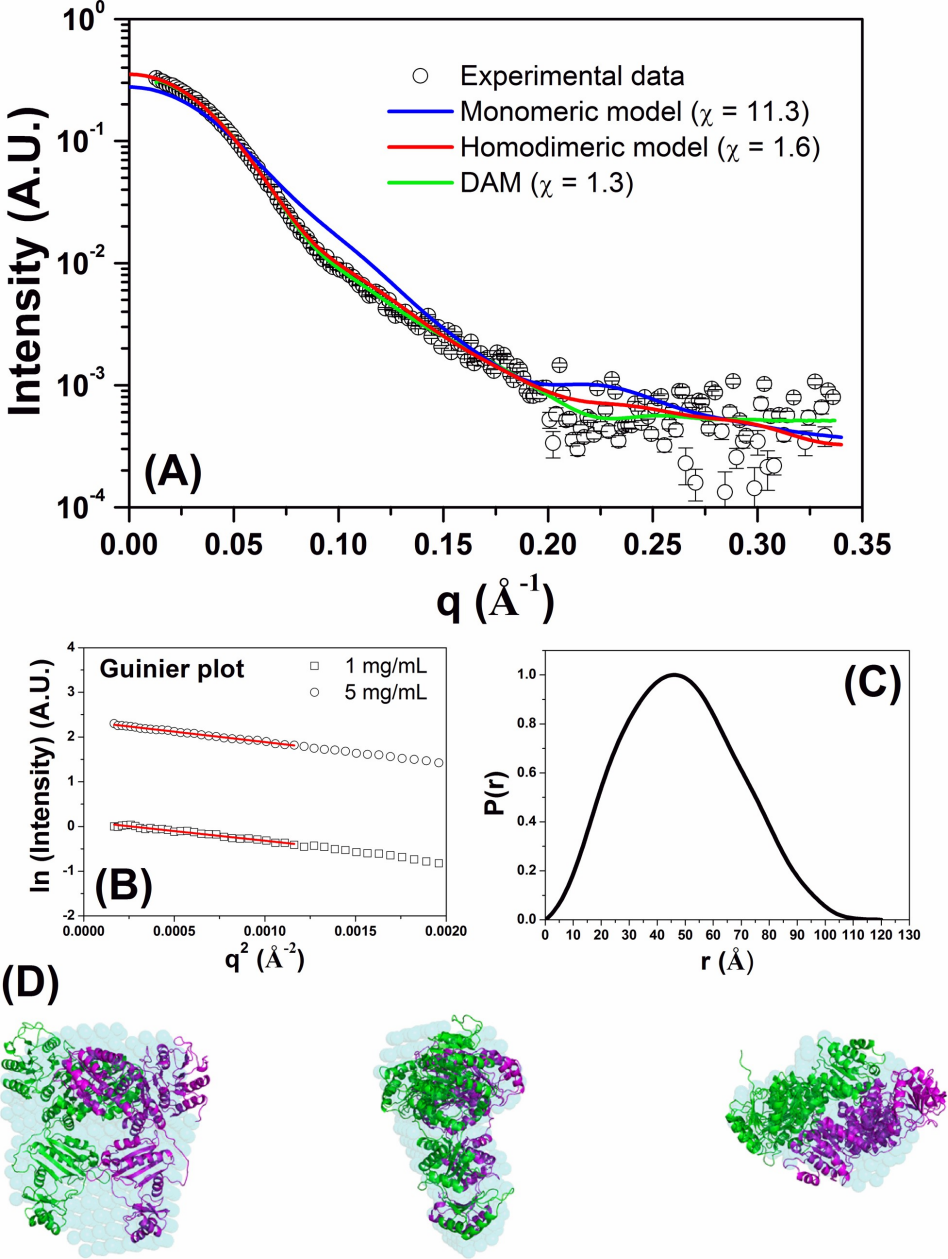
SAXS data measured for FCS1 at pH 7.0 and 20 ^o^C. (**A**) Experimental SAXS curve (open black circles with errors bars) superimposed on the theoretical SAXS curves based on the monomeric (red line) and homodimeric (blue line) homology models. The green line was determined based on the low-resolution model. (**B**) Guinier plots at 1 and 5 mg/mL. (**C**) Experimental distance distribution function, P(r), of FCS1 at 20 ^o^C. The curve has been scaled to a maximum height of 1. (**D**) Superposition of the low-resolution envelope of FCS1 (obtained by DAMMIN program) on the homodimeric homology model shown in three different views (the center and right structures were rotated y axis-90^o^ and x axis-90^o^ in relation to the left structure).

The SAXS analyses were performed to obtain further information about the tertiary/quaternary structure of FCS1 and its low-resolution envelope in solution. The X-ray scattering curve measured at 5 mg/mL for FCS1 (at pH 7 and 20 ^o^C) is shown in Fig 5A. The Guinier plots (at 1 and 5 mg/mL) showed linear behaviors indicating excellent monodispersity (Fig 5B). The radius of gyration (R_g_) determined with the Guinier approximation at 1 and 5 mg/mL were 36.4 ± 1.1 Å and 37.4 ± 0.5 Å, respectively. The distance distribution function (at 5 mg/mL) determined using the GNOM software (Fig 5C) estimated the maximum dimension (D_max_) and R_g_ as being 120 ± 5 Å and 37.2 ± 0.1 Å, respectively. The monomeric FCS1 homology model has a D_max_ and R_g_ of 110.1 Å and 34.4 Å, respectively, values different from those determined by SAXS analysis. This explains why the theoretical SAXS curve based on the monomeric FCS1 homology model does not fit very well (χ = 11.3) in the SAXS data (blue line, Fig 5A). However, the values determined for homodimeric FCS1 homology model (D_max_ = 128.5 Å and R_g_ = 37.5 Å) are in agreement with the results obtained by SAXS analysis. The theoretical SAXS curve calculated from the homodimeric FCS1 homology model resulted in an excellent fit (χ = 1.6, red line) to SAXS data. In each case, the quality of the fitting can be observed by the residual plots, where the experimental intensity divided by computed intensity was plotted as a function of the scattering vector q (S7 Fig). Therefore, the SAXS data for FCS1 is consistent with a homodimeric protein in solution arranged with a twofold symmetry [53]. The low-resolution envelope of FCS1 was determined directly in solution from the SAXS data (Fig 5D). Superposition of the low-resolution envelope and homodimeric FCS1 homology model showed excellent agreement. A summary of the main SAXS data described in this study is given in Table 2.

**Table 2:** General SAXS results from FCS1.

Parameters	Experimental data	PDB	Model Monomer	Model Homodimer	DAM
R_g_ (Å)	36.4 ±1.1 (1 mg/mL) 37.4±0.5 (5 mg/mL) 37.2 ±- 0.1 (GNOM)	37.5	34.4	37.5	37.3
D_max_ (Å)	120 ± 5	126.6	110.1	128.5	119.1
Resolution (Å)	18.5	-	-	-	18.5
χ	-	1.9	11.3	1.6	1.3

### The pH induced conformational changes in the three domains of FCS1

**Fig 6:**
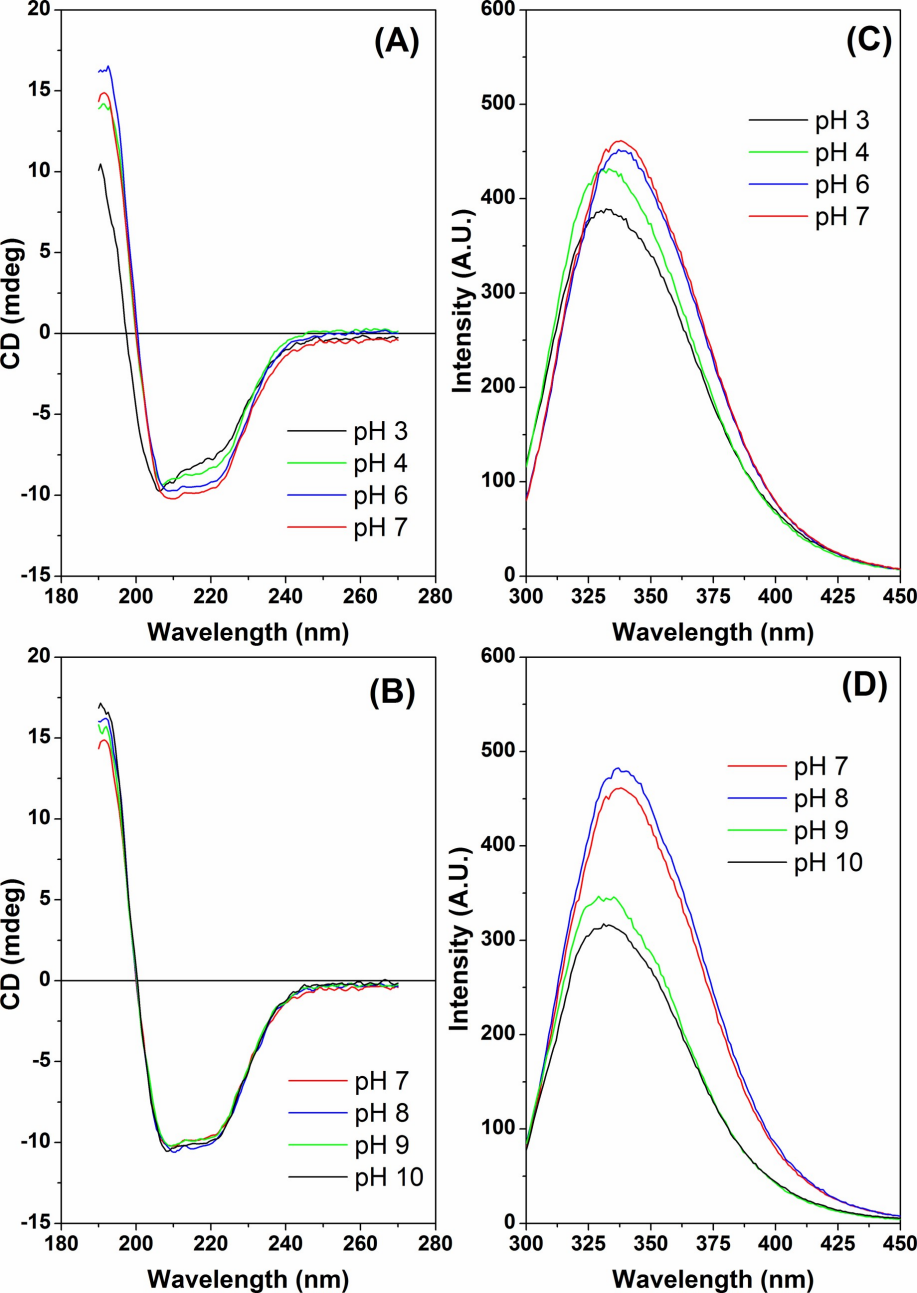
Effect of pH on the secondary and tertiary structures of FCS1 at 20°C (**A**) CD spectra as a function of pH. The pH values were 3.0, 4.0, 6.0 and 7.0. (**B**) CD spectra as a function of pH. The pH values were 7.0, 8.0, 9.0 and 10.0. (**C**) Effect of pH on the tertiary structure of FCS1 monitored by intrinsic fluorescence spectroscopy. The pH values were 3.0, 4.0, 6.0 and 7.0. (**D**) Effect of pH on the tertiary structure of FCS1 monitored by intrinsic fluorescence spectroscopy. The pH values were 7.0, 8.0, 9.0, 10.0.

Circular dichroism (CD) spectroscopy was employed to analyze the secondary structure of the FCS1 in response to pH and temperature. Fig 6A and 6B represents the CD spectra of the FCS1 measured at different pH values at 20 ^o^C. At pH 7.0 (red line), the CD spectrum of FCS1 was characterized by two minima at 208 and 220 nm, a maximum around 193 nm, and a negative to positive crossover at 200 nm. The spectrum is characteristic of an α/β protein, where the two minima at 208 nm and 220 nm are indicative of the presence of α-helical secondary structure. When the pH value was decreased from 7.0 to 3.0 (Fig 6A), changes were observed in the profiles of the spectra (mainly at pH 3.0 and 4.0) indicating a pH-induced loss of regular secondary structures at acidic pH values, in agreement with the marked decrease of the enzymatic activity described above. At pH 5.0 (theoretical pI value) the protein precipitated irreversibly after overnight incubation. However, when the pH value was increased from 7.0 to 10.0, no significant changes were observed in the profiles of the spectra indicating stability of second structure under alkaline pH (Fig 6B), which corroborates with the biochemical data described above.

**Fig 7:**
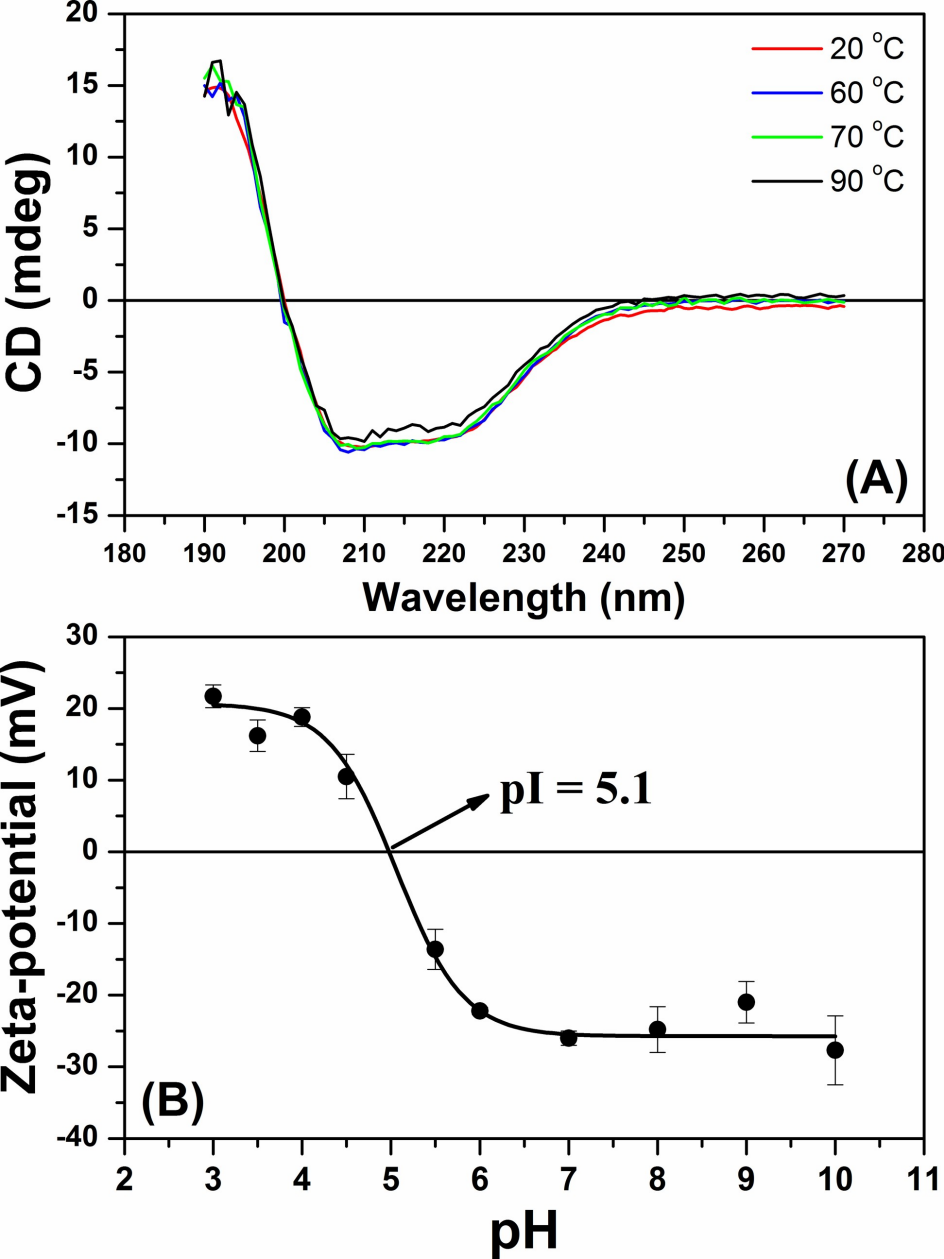
(**A**) Effect of temperature (20, 60, 70 and 90°C) on the secondary structure of FCS1 at pH 7.0. (**B**) FCS1 zeta-potential (ζ) values as a function of pH variation.

The secondary structure of FCS1 was also analyzed at different temperatures, as shown in Fig 7. Even at 70 ^o^C the spectrum profile did not change significantly, indicating that the FCS1 secondary structure is thermostable. However, significant changes in the spectrum profile were observed when the protein was incubated at 90 ^o^C, indicating partial loss of the regular secondary structures.

The presence of three tryptophan residues (W212, W450 and W604) was used to monitor FCS1 conformational changes induced by intrinsic fluorescence spectroscopy. The tryptophan fluorescence emission can be selectively excited at 295 nm. Fig 6C and 6D show the fluorescence emission spectra of FCS1 at different pH values. At pH 6.0, 7.0 and 8.0, the spectra were characterized by a maximum emission at 338 nm, typical of tryptophan residues partially exposed to buffer environment. However, at pH values smaller than 6.0 and greater than 8.0, the spectra were characterized by a maximum emission at 332 nm, indicating that, at these pH values, the tryptophan residues are more buried inside the protein structure when compared to pHs 6.0, 7.0 and 8.0. Collectively, these pH-induced conformational changes corroborate the activity data at different pHs (Fig 4B) and functional stability of enzyme activity under alkaline conditions (Fig 4C).

As mentioned previously, FCS1 presents a highly conserved histidine residue (His267), which has been shown to be transiently phosphorylated during catalysis in succinyl-CoA synthetase from *Escherichia coli* and ADP-forming acetyl-CoA synthetase from *Pyrococcus furiosus* [63,65]. This mechanism of intermediate catalysis is favored by the nature of phosphohistidines, which contain relatively unstable phosphoramidate bonds at nitrogen atoms, as opposed to other phosphoamino acids that present more stable phosphoester bonds. In general, phosphoramidate bonds are particularly prone to degradation at acidic pH values, while remaining stable in basic environments [78]. This chemical behavior could explain FCS1 poor activity at acid pH values and relatively high activity at alkaline pH values, which is in accordance with the conformational changes here presented.

Finally, the zeta-potential (ζ) values as a function of pH were determined for FCS1 (Fig 7B). At pH 3.0 the ζ was 21.7 ± 1.6 mV and decreased for -27.7 ± 4.8 mV at pH 10.0. The experimental isoelectric point (pI) was estimated to be 5.1, consistent with the theoretical pI (pI = 5.03, predicted by ProtParam tool). An abrupt variation in the ζ was observed between pH 6 and 3 (acid pH values), resulting in repulsion among charged groups that causes the significant conformational modifications (in both secondary and tertiary structures) described above and affecting directly the enzymatic activity [62]. However, at alkaline pH values the ζ varies subtly, thus corroborating with the variation in conformation and enzymatic activity described previously.

## Conclusion

The present study brings to light as the first biophysical and structural characterization of a bacterial Fcs, a class of enzymes of pivotal importance for lignin valorization via conversion of FA. The FCS1, herein described, was isolated from a lignin-degrading microbial consortium, denoting its importance in an ecological context as well. Albeit important, there are few thorough characterizations of Fcs in the literature. The FCS1 enzymatic activity remains high in a range of mesophilic temperatures, as well as neutral and alkaline pH values. We have determined that FCS1 constitutes a homodimer in solution, which is in agreement to the 3D-architecture of the closest homologue of known structure. The high activity of FCS1 in higher pH values could be useful for direct conversion of biorefinery derived lignin-streams, which are usually obtained via alkaline treatment. Finally, this study could contribute to the field of research by establishing a structural and biochemical characterization for Fcs.

## Supporting information

S1 Fig The proposed catalytic activity of Fcs from FA. (TIF)

S2 Fig (A) HisTrapTMHP affinity chromatograpy profile. Green line indicates the linear gradient of buffer B (20 mM sodium phosphate buffer pH 7.0, 100 mM NaCl, 500 mM imidazole). Fractions 1 and 2 marked as red numbers were collected to further purification in size-exclusion chromatography. (B) Superdex 200 HiLoad 16/600 GL size exclusion chromatography. Elution was performed in buffer C (20 mM sodium phosphate buffer pH 7.4, 100 mM NaCl).
(TIF)

S3 Fig Capillary zone electrophoresis of FCS1 enzymatic products at different alkaline pHs. Enzymatic reactions contained 1 mM FA and 10 μg purified enzyme. After incubation for 5 minutes at 37°C, the reactions were immediately diluted 1:10 in methanol 100%. All the measurements were made with the P/ACE MDQ capillary electrophoresis system (Beckman Coulter Inc., USA) equipped with an UV detector. Fused-silica capillaries with inner diameter 50 μm, outer diameter 365 μm, and total length 35.5 cm (25 cm to the detector) were used. Capillaries were conditioned with reagents supplied by the Capillary Performance Test Kit (Beckman Coulter Inc., USA). Before and after use, the capillary was rinsed with: Regenerator solution A (10 min, 25.0 psi), Milli-Q purified water (2 min, 25.0 psi) and Capillary Performance Run Buffer A (4 min, 25.0 psi). Between analyses, the capillar was conditioned with Regenerator A (2 min, 25.0 psi), Milli-Q water (1 min, 25.0 psi) and Performance Buffer A (3 min, 25.0 psi). Samples were injected for 10 sec, 0.5 psi. Voltage (+25 kV) was then applied for 10 min. Data were collected and processed with 32Karat software (Beckmann Coulter Inc., Fullerton, CA, USA).
(TIF)

S4 Fig Spectral scanning of FA in different buffer systems. (A) 100 mM potassium phosphate buffer. (B) 20 mM ABF buffer. (C) Difference of absorbances in potassium phosphate and ABF buffers. 0.5 mM of FA was mixed with either 20 mM ABF buffer or 100 mM potassium phosphate buffer, pHs 5.8, 6.2, 6.6, 7.0, 7.4, 7.8, 8.0, 8.2, 8.6, 9.0, 9.4 and 9.8, in a final volume of 200 μL. The mixtures were placed in a 3mm quartz cuvette and the absorbance in wavelengths from 200 nm to 700 nm was read using and Epoch2 Microplate Reader spectrophotometer (BioTek, Winooski, VT, USA).
(TIF)

S5 Fig FCS1 kinetics (Michaelis-Menten plot). (TIF)

S6 Fig Determination of FCS1 molecular weight. (**A**) Size exclusion chromatography (SEC) of purified FCS1 on Superdex-200. (**B**) Size distribution by intensity for purified FCS1 where dynamic light scattering (DLS) runs were performed at pH 7.0 and 20 ^o^C.
(TIF)

S7 Fig Residual plots where the experimental intensity divided by computed intensity was plotted as a function of the scattering vector q. (**A**) Monomeric model. (B) Homodimeric model. (**C**) Dummy Atom Model (DAM).
(TIF)
