# Supplementary figures and images for: An alkaline active feruloyl-CoA synthetase from soil metagenome as a potential key enzyme for lignin valorization strategies
Source: PLoS One. 2019 Feb 25;14(2):e0212629. doi: 10.1371/journal.pone.0212629 (PMC6388921; doi:10.1371/journal.pone.0212629)

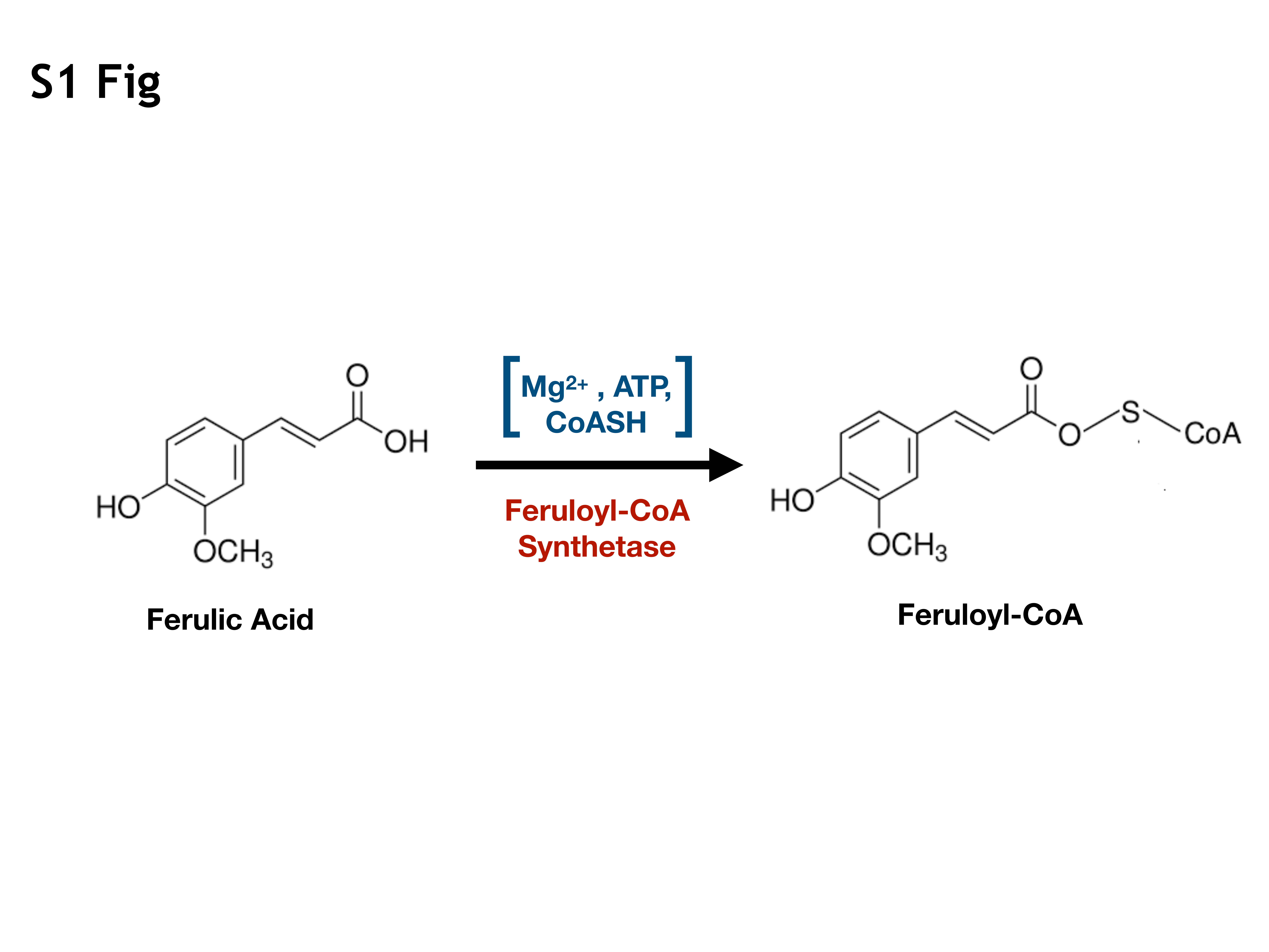

Supplement: S1 Fig — (TIF) [file pone.0212629.s001.tif]

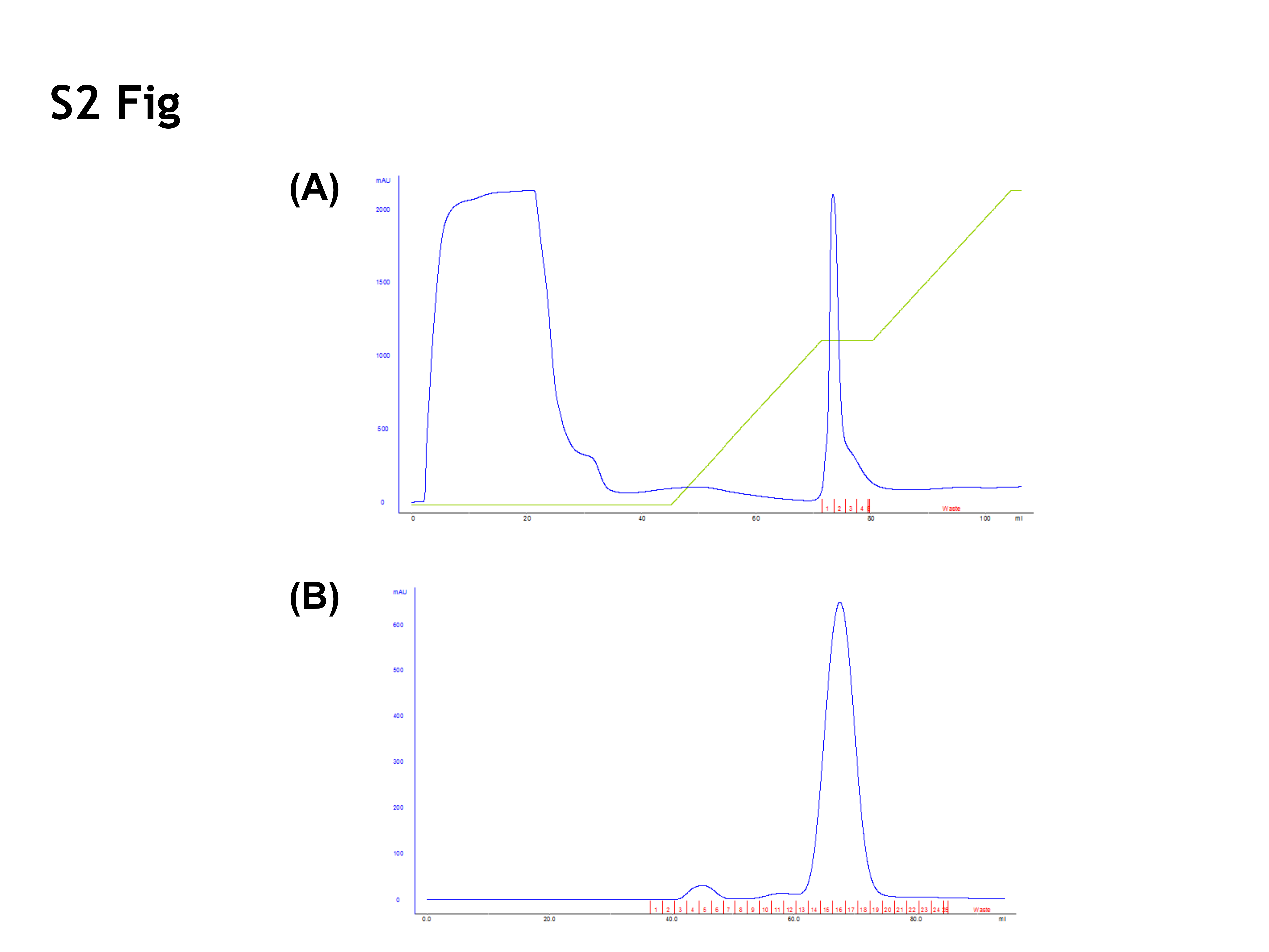

Supplement: S2 Fig — (A) HisTrapTMHP affinity chromatograpy profile. Green line indicates the linear gradient of buffer B (20 mM sodium phosphate buffer pH 7.0, 100 mM NaCl, 500 mM imidazole). Fractions 1 and 2 marked as red numbers were collected to further purification in size-exclusion chromatography. (B) Superdex 200 HiLoad 16/600 GL size exclusion chromatography. Elution was performed in buffer C (20 mM sodium phosphate buffer pH 7.4, 100 mM NaCl). (TIF) [file pone.0212629.s002.tif]

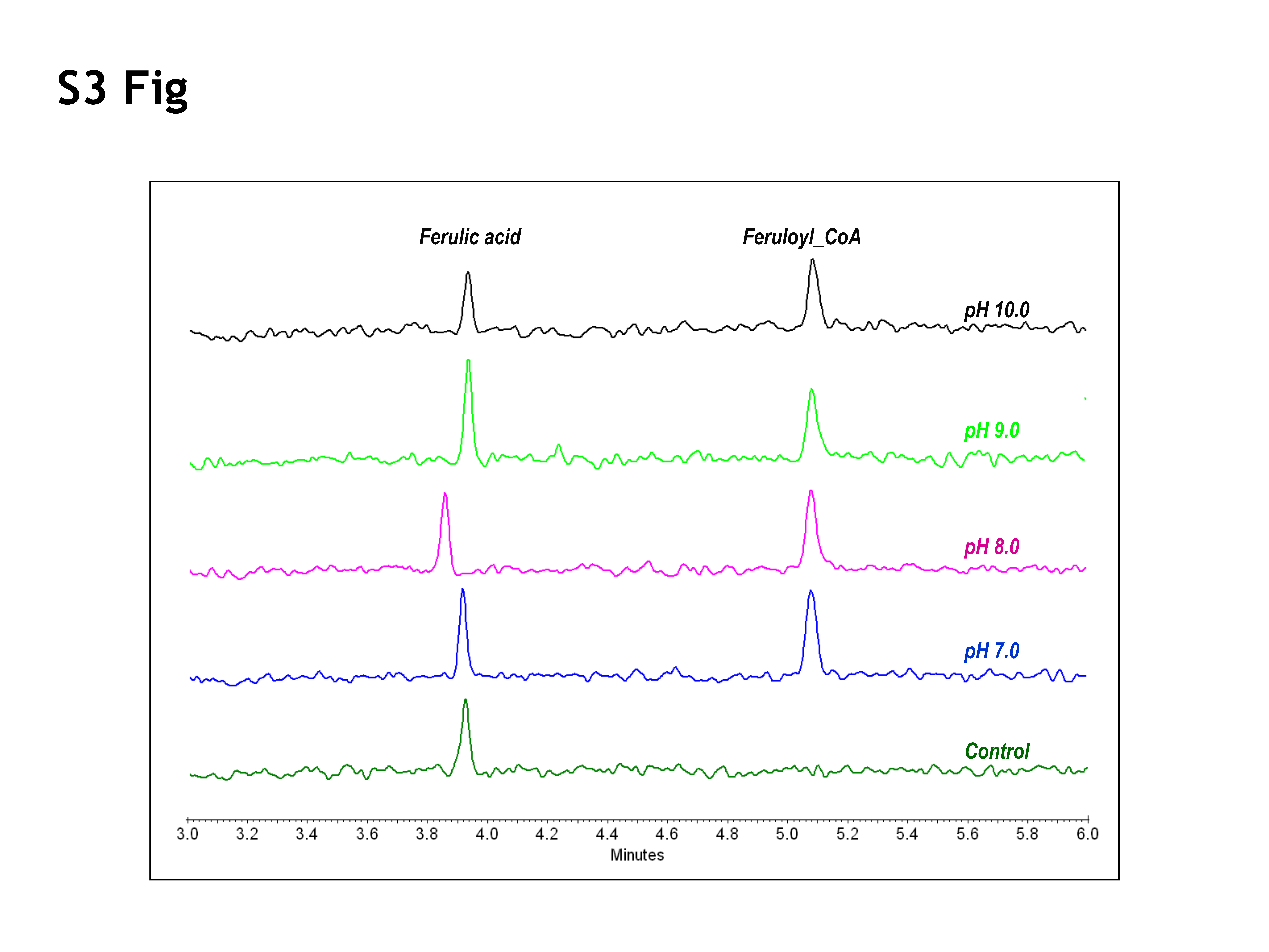

Supplement: S3 Fig — Enzymatic reactions contained 1 mM FA and 10 μg purified enzyme. After incubation for 5 minutes at 37°C, the reactions were immediately diluted 1:10 in methanol 100%. All the measurements were made with the P/ACE MDQ capillary electrophoresis system (Beckman Coulter Inc., USA) equipped with an UV detector. Fused-silica capillaries with inner diameter 50 μm, outer diameter 365 μm, and total length 35.5 cm (25 cm to the detector) were used. Capillaries were conditioned with reagents supplied by the Capillary Performance Test Kit (Beckman Coulter Inc., USA). Before and after use, the capillary was rinsed with: Regenerator solution A (10 min, 25.0 psi), Milli-Q purified water (2 min, 25.0 psi) and Capillary Performance Run Buffer A (4 min, 25.0 psi). Between analyses, the capillar was conditioned with Regenerator A (2 min, 25.0 psi), Milli-Q water (1 min, 25.0 psi) and Performance Buffer A (3 min, 25.0 psi). Samples were injected for 10 sec, 0.5 psi. Voltage (+25 kV) was then applied for 10 min. Data were collected and processed with 32Karat software (Beckmann Coulter Inc., Fullerton, CA, USA). (TIF) [file pone.0212629.s003.tif]

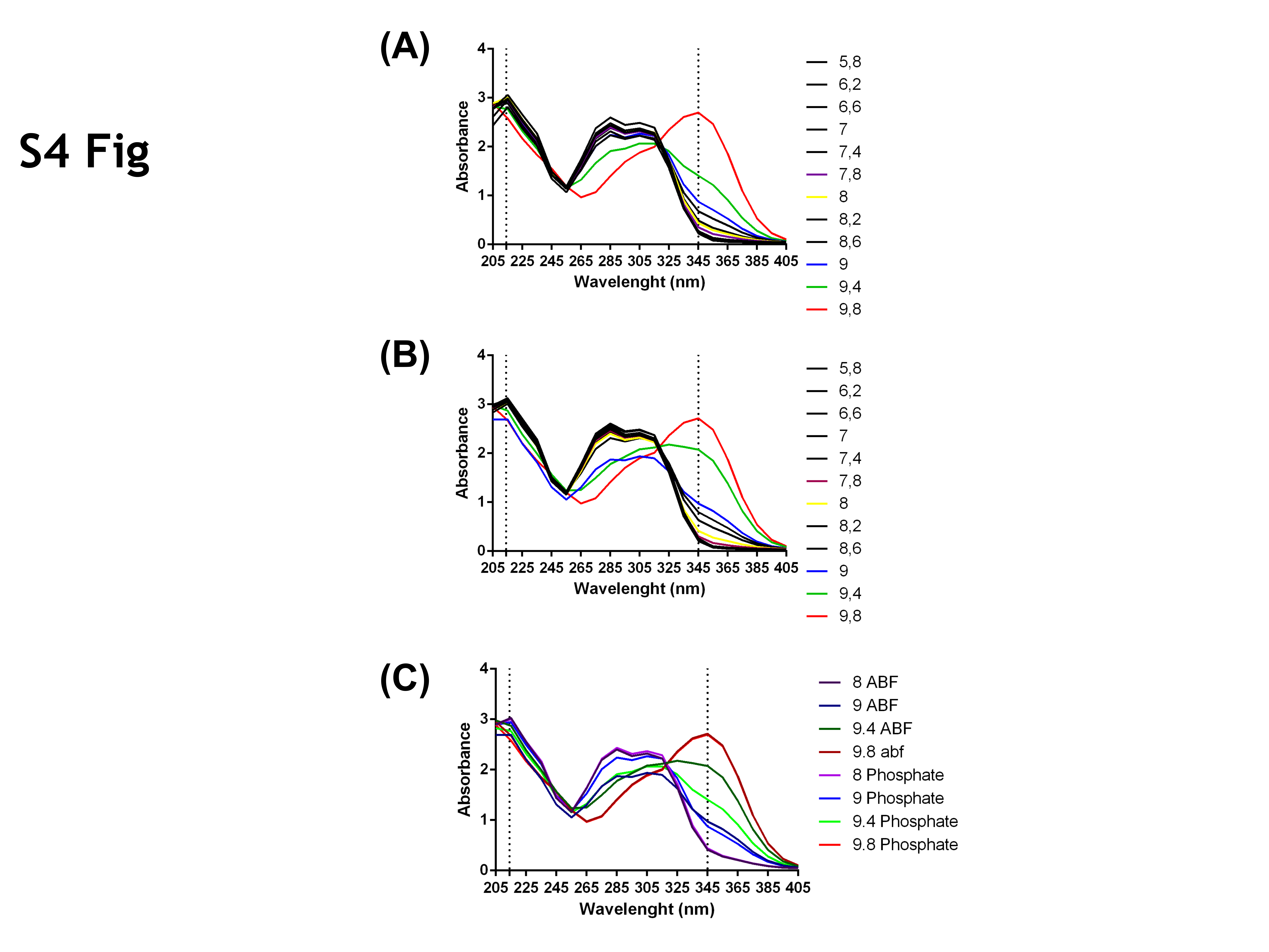

Supplement: S4 Fig — (A) 100 mM potassium phosphate buffer. (B) 20 mM ABF buffer. (C) Difference of absorbances in potassium phosphate and ABF buffers. 0.5 mM of FA was mixed with either 20 mM ABF buffer or 100 mM potassium phosphate buffer, pHs 5.8, 6.2, 6.6, 7.0, 7.4, 7.8, 8.0, 8.2, 8.6, 9.0, 9.4 and 9.8, in a final volume of 200 μL. The mixtures were placed in a 3mm quartz cuvette and the absorbance in wavelengths from 200 nm to 700 nm was read using and Epoch2 Microplate Reader spectrophotometer (BioTek, Winooski, VT, USA). (TIF) [file pone.0212629.s004.tif]

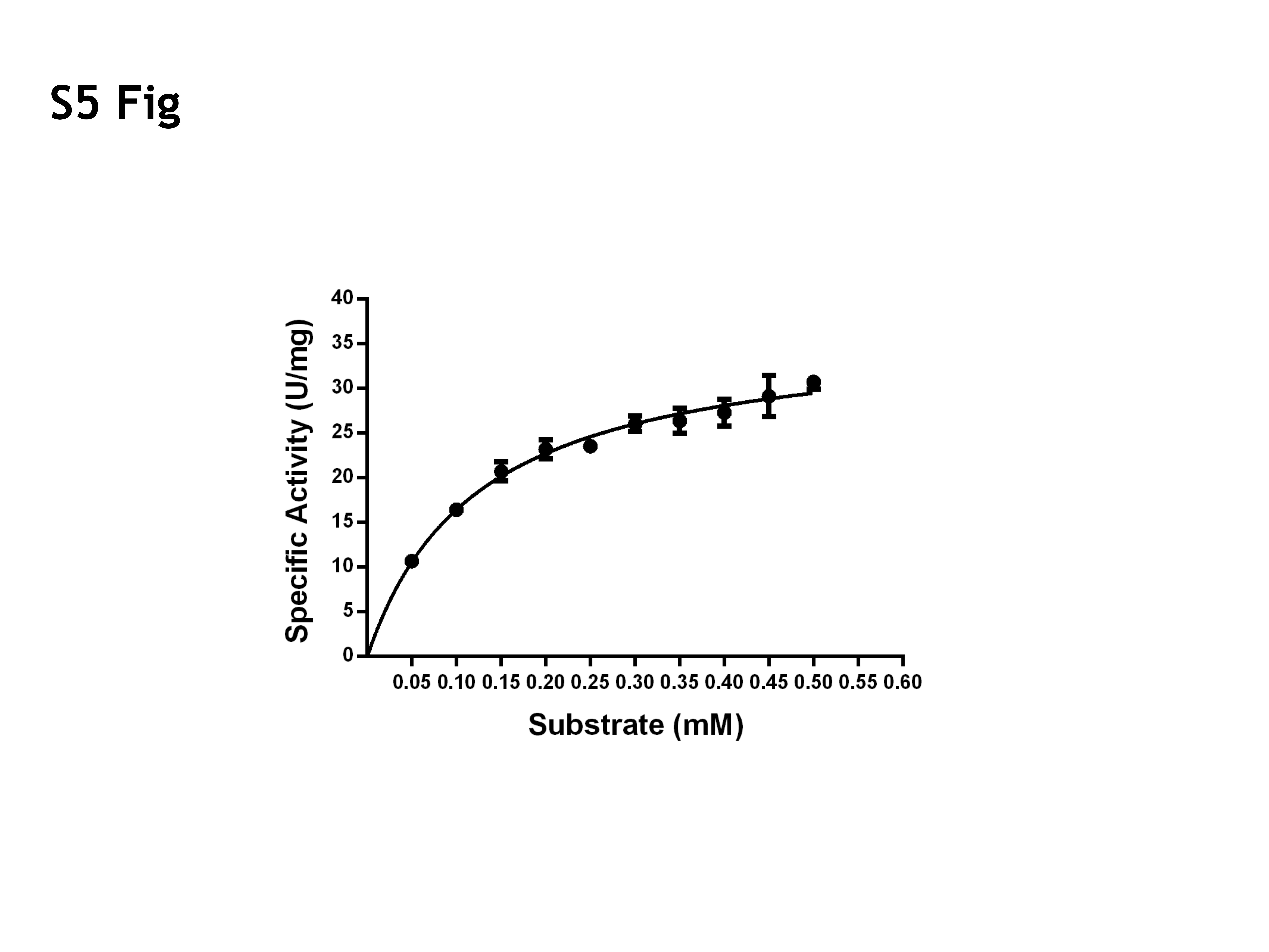

Supplement: S5 Fig — (TIF) [file pone.0212629.s005.tif]

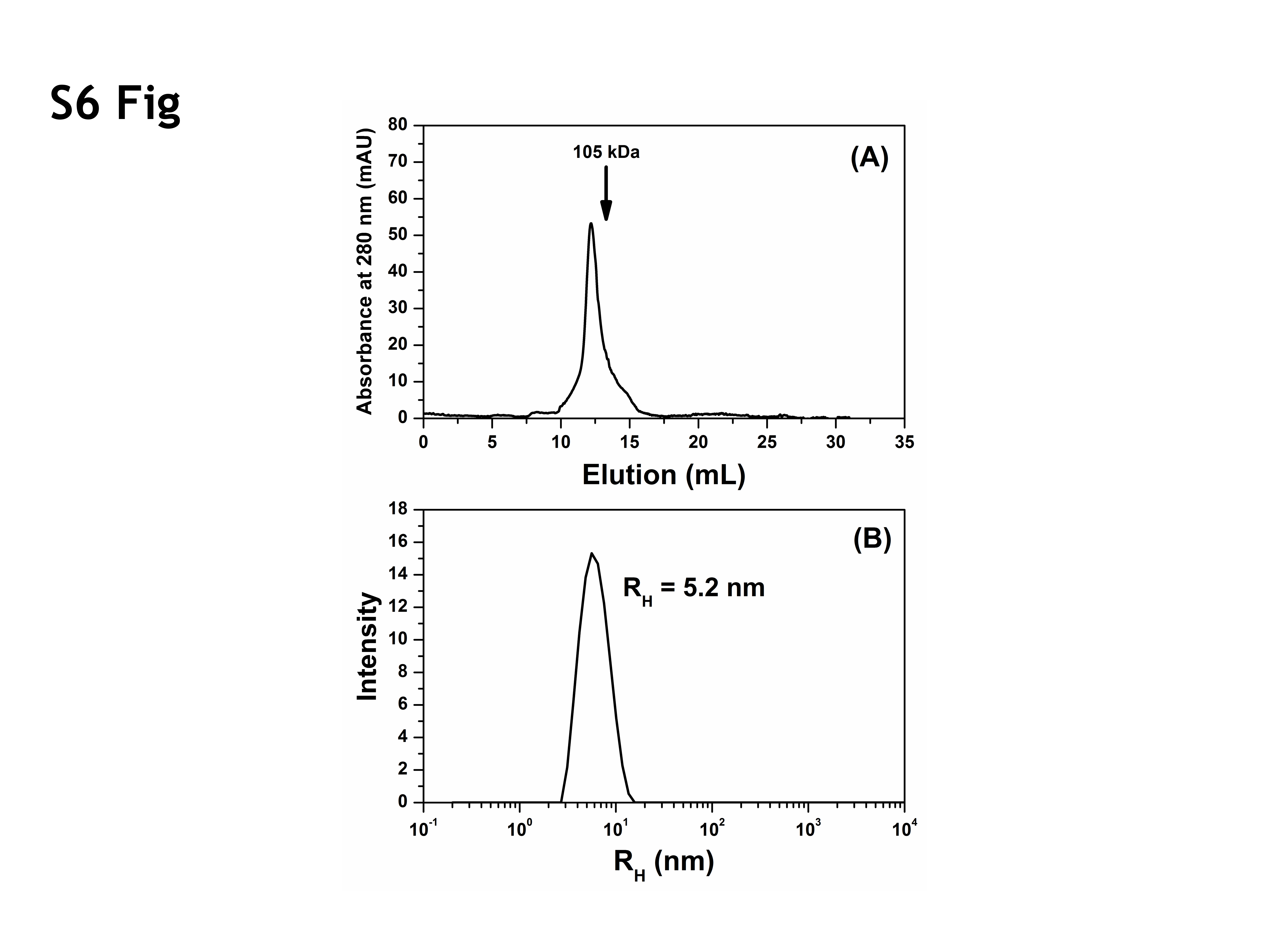

Supplement: S6 Fig — (A) Size exclusion chromatography (SEC) of purified FCS1 on Superdex-200. (B) Size distribution by intensity for purified FCS1 where dynamic light scattering (DLS) runs were performed at pH 7.0 and 20 oC. (TIF) [file pone.0212629.s006.tif]

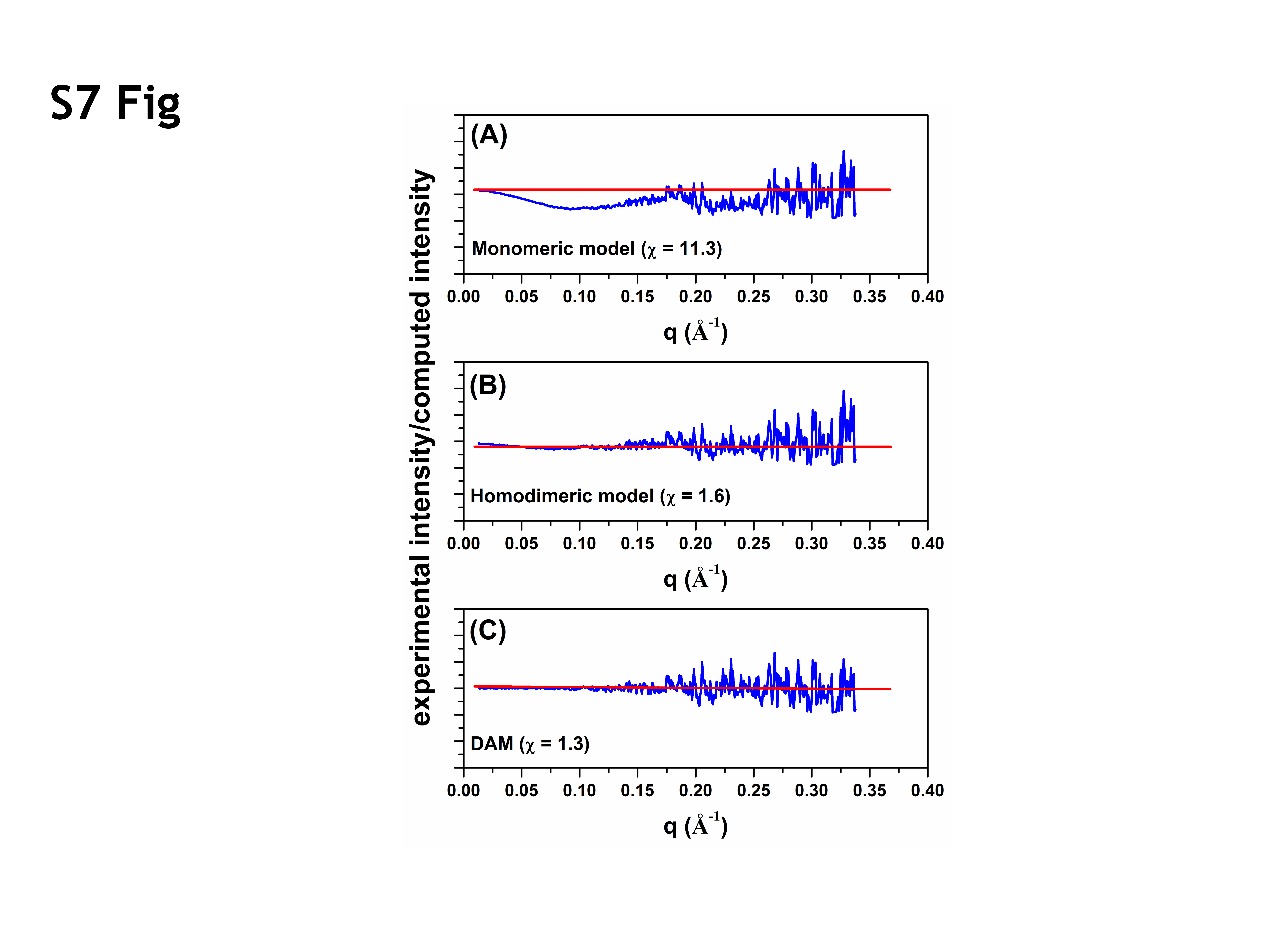

Supplement: S7 Fig — (A) Monomeric model. (B) Homodimeric model. (C) Dummy Atom Model (DAM). (TIF) [file pone.0212629.s007.tif]
